# Foley Catheter and Islamic Rulings on Purification: A Clinical–Fiqh Review and Patient–Community Survey

**DOI:** 10.12669/pjms.42.8.15640

**Published:** 2026-08

**Authors:** Wajeed Gul Bangash, Muhammad Ghayas, Ubaid Ullah Sindhi, Abdul Razaq Khan

**Affiliations:** 1 Wajeed Gul Bangash, MBBS, MS(Urology) Department of Urology, Pakistan Railways General Hospital, Rawalpindi, Pakistan; 2 Muhammad Ghayas, PhD, Department of Islamic Studies, Riphah International University, Islamabad, Pakistan; 3 Ubaid Ullah Sindhi, PhD Department of Islamic Studies, Bahria University Islamabad, Islamabad, Pakistan; 4 Abdul Razaq Khan, PhD, Department of Islamic Studies, Riphah International University, Islamabad, Pakistan

**Keywords:** Foley catheter, Islamic rulings, Ritual purification, Wudu, Tayammum, Patient counseling, Urology

## Abstract

**Background & Objective:**

Foley and suprapubic catheters are commonly used for postoperative urinary drainage and intake–output monitoring. For practicing Muslims, continuous urinary drainage raises important concerns regarding ritual purification (wuḍū), prayer validity, and recitation of the Qur’an. Our objective was to review Islamic jurisprudential (fiqh) rulings related to catheterized patients and to assess awareness, concerns, and acceptance of these rulings among patients and the wider community, with the aim of providing practical clinical guidance.

**Methodology:**

This hospital based mixed-method study comprised: (1) a clinical–fiqh review of classical and contemporary Islamic rulings regarding purification in patients with continuous urinary drainage, and (2) a structured questionnaire-based survey conducted among catheterized patients and community participants. The study was conducted jointly at the Department of Urology, Pakistan Railways General Hospital, Rawalpindi, and the Department of Islamic Studies, Riphah International University, Islamabad, from January to June 2024. A bilingual (English–Urdu) questionnaire was administered before and after counseling. This assessment was designed to evaluate the clinical impact of a structured, faith-sensitive counseling intervention delivered as part of routine patient care, rather than to conduct a standalone knowledge, attitude, or practice survey.

**Results:**

A total of 500 participants were enrolled, including 250 catheterized patients and 250 community members. Baseline awareness of Islamic rulings related to catheterization was 22%, which increased to 91% following counseling (p < 0.001). Most participants reported fear of missing prayers or reciting the Qur’an incorrectly. Jurisprudential review demonstrated consensus that catheterized patients are classified as *ma‘dhūr* (legally excused) and may perform prayer according to prescribed concessions.

**Conclusion:**

Catheterized Muslim patients should be counseled in accordance with established Islamic rulings on purification to prevent unnecessary spiritual distress. Integrating clinical care with fiqh-based guidance supports holistic patient well-being without compromising medical management.

## INTRODUCTION

The human body produces several forms of excreta. Among these, urine holds particular medical and religious significance. Medically, urine represents a metabolic waste product excreted by the kidneys, while in Islamic jurisprudence it is classified as *najis* (ritually impure), necessitating purification before acts of worship such as prayer and recitation of the Qur’an. Avoidance of urine contamination and appropriate purification through *istinjā’*, wuḍūʾ, and clean clothing are essential requirements for the validity of prayer, reflecting Islam’s emphasis on both physical hygiene and spiritual cleanliness.

Islamic teachings strongly emphasize cleanliness and protection from urine contamination. The Qur’an states: *“And purify your clothing”* (Qur’an, 74:4),^1^ underscoring the obligation of maintaining physical purity during worship. Similarly, the Prophet Muhammad (peace be upon him) warned against negligence in this regard, stating: *“They are being punished in their graves; one of them used not to protect himself from his urine”* (Ṣaḥīḥ al-Bukhārī; Ṣaḥīḥ Muslim).^2^,^3^ These foundational texts shape Muslim patients’ understanding of ritual purity and contribute to heightened concern when urinary control is compromised.

Urinary catheters, including Foley and suprapubic catheters, are widely used in urological and surgical practice for postoperative drainage, chronic urinary retention, and accurate intake–output monitoring. While medically indispensable, the presence of an indwelling catheter introduces continuous and involuntary urinary drainage, creating uncertainty among Muslim patients regarding the validity of wuḍūʾ, prayer, and Qur’an recitation. In Islamic jurisprudence, continuous urinary leakage resembles the classical condition of urinary incontinence (*salas al-bawl*), for which specific legal concessions exist.

Islamic jurisprudence addresses such circumstances by classifying affected individuals as *ma‘dhūr* (legally excused), allowing modified rulings that preserve religious obligations without imposing undue hardship. Under these rulings, patients may perform purification according to prescribed concessions and continue prayer despite ongoing urinary flow. However, these principles are not universally understood by patients or the general community, particularly in hospital settings where religious counseling is often absent.

Despite the availability of clear fiqh principles, many hospitalized Muslim patients report confusion, anxiety, and even omission of prayer during catheterization due to fear of invalid worship. This concern was repeatedly expressed by patients at Pakistan Railways General Hospital, Rawalpindi, highlighting a gap between jurisprudential knowledge and clinical practice. Failure to address such concerns may adversely affect patients’ psychological and spiritual well-being, an increasingly recognized component of holistic healthcare.

### Therefore, the present study was undertaken with two primary objectives:

- To synthesize classical and contemporary Islamic rulings related to purification in catheterized patients into a clinically applicable framework.
- To assess awareness, concerns, and acceptance of these rulings among catheterized patients and the broader community through a structured survey.

## METHODOLOGY

This prospective hospital based mixed-method study was conducted collaboratively by the Department of Islamic Studies, Riphah International University, Islamabad, and the Department of Urology, Pakistan Railways General Hospital, Rawalpindi, over a six-month period from January to June 2024.

### Ethical Approval:

The study was approved by the relevant institutional ethical review authorities prior to data collection, and all participants provided written informed consent. Ref. No .Riphah/DIS/REC/25/01 Dated March 12^th^ 2025.

The study population comprised two distinct groups: adult Muslim patients (≥18 years) with indwelling Foley or suprapubic catheters in situ for more than 24 hours, and healthy community volunteers recruited from outpatient attendants and local community groups. Inclusion criteria were Muslim faith, age ≥18 years, and willingness to participate. Exclusion criteria included non-Muslims, individuals younger than 18 years, and patients who were critically ill, unconscious, or cognitively impaired and therefore unable to provide informed consent.

A total of 500 participants were enrolled, including 250 catheterized patients and 250 community members. The sample size was determined pragmatically based on patient availability during the study period and feasibility considerations. Data were collected using a structured bilingual (English–Urdu) questionnaire developed through expert consultation with urologists and Islamic scholars to ensure both clinical relevance and religious accuracy. The questionnaire assessed demographic characteristics, baseline knowledge and beliefs regarding ritual purification (wuḍūūʾand *tayammum*) in the presence of urinary catheterization, concerns related to prayer continuity, and levels of confidence and spiritual reassurance.

Each participant completed the questionnaire twice: once prior to counseling and once following a standardized counseling session. The counseling intervention consisted of brief, face-to-face guidance integrating basic clinical explanations of catheter function with established Islamic jurisprudential principles relevant to purification and prayer during continuous urinary drainage. The questionnaire was employed as an outcome assessment tool to evaluate the effect of a structured, faith-sensitive counseling intervention integrated into routine clinical care, rather than as an *independent knowledge, attitude, or practice (KAP) survey*.

### Statistical Analysis:

Data were entered and analyzed using Statistical Package for the Social Sciences (SPSS) software. Descriptive statistics were used to summarize participant characteristics and response frequencies. Pre- and post-counseling changes in awareness and attitudes were assessed, with a p-value of <0.05 considered statistically significant.

## RESULTS

### Demographic characteristics:

A total of 500 participants were included in the study, comprising 250 catheterized patients and 250 community members. The mean age of participants was 54 ± 12 years (range: 18–80 years). Overall, 62% of participants were male and 38% were female. Approximately 40% of participants reported no formal education.

**Table-I:** Awareness and concerns of participants before counseling (n = 500).

Variable	n (%)
Correct knowledge of ruling	110 (22%)
Fear of missing prayer	325 (65%)
Fear of Qur’an recitation without purity	290 (58%)

*This table summarizes baseline knowledge of purification rulings and common religious concerns reported by participants prior to counseling.*

### Awareness and concerns prior to counseling:

Baseline assessment revealed limited awareness regarding Islamic rulings related to purification in catheterized patients. Only 110 (22%) participants demonstrated correct knowledge of the relevant rulings. Fear of missing obligatory prayers was reported by 325 (65%) participants, while 290 (58%) expressed concern about reciting the Qur’an without adequate purification.

### Impact of counseling on awareness and attitudes:

Following structured clinical–fiqh counseling, a marked improvement was observed in participants’ understanding and confidence. Correct knowledge of purification rulings increased from 22% to 91%, which was statistically significant (p < 0.001). In addition, 435 (87%) participants reported improved spiritual reassurance, and 460 (92%) recommended wider dissemination of this guidance.

**Fig.1:**
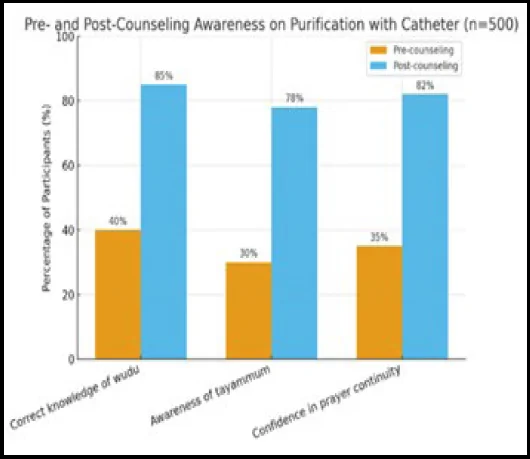
Pre- and post-counseling awareness regarding purification and prayer continuity. *The bar chart illustrates improvements in correct knowledge of* wuḍūʾ*, awareness of tayammum, and confidence in maintaining prayer continuity among participants following counseling (p < 0.001). which demonstrates substantial improvements in correct knowledge of* wuḍūʾ*, awareness of tayammum, and confidence in maintaining prayer continuity during catheterization*.

**Table-II:** Post-counseling improvement in awareness and attitudes (n = 500).

Variable	n (%)	p-value
Correct knowledge of ruling	455 (91%)	<0.001
Reported spiritual peace	435 (87%)	–
Recommended sharing guidance widely	460 (92%)	–

*This table presents changes in awareness, spiritual reassurance, and attitudes following structured counseling, including statistical significance where applicable. Changes in awareness and confidence before and after counseling are illustrated in Fig.1.*

## DISCUSSION

The Qur’anic principle that *“Allah does not burden a soul beyond that it can bear”* (Qur’an 2:286)^4^ provides a foundational framework for understanding religious concessions during illness. In the context of medical care, this principle emphasizes ease, mercy, and practicality rather than hardship. The present study demonstrates that structured counseling based on Islamic jurisprudence significantly improves awareness, confidence, and spiritual reassurance among catheterized patients and community members.

Our findings highlight that uncertainty regarding ritual purification and prayer validity creates substantial psychological distress. Many participants reported that they were willing to endure physical discomfort but not religious ambiguity, underscoring the importance of faith-sensitive counseling within hospital settings. The inclusion of community participants further revealed a broader knowledge gap, indicating that awareness initiatives should extend beyond hospitals to mosques and community platforms.

Comparable to the work of Pallathadka et al,^5^ who emphasized the role of Islamic teachings in promoting ethical conduct, our study extends the application of Qur’anic guidance into clinical practice. While their work focused on education, the present study applies similar principles to urology, particularly addressing patient dignity and religious continuity during Foley catheterization.

In contrast, Saruhan et al.^6^ examined the perception that Islam instills fear through concepts of punishment. Their analysis clarified those Islamic teachings balance accountability with mercy and hope. Our findings align with this perspective by illustrating that Islamic jurisprudence does not impose unnecessary hardship on patients. The classification of catheterized patients as *ma‘dhūr* (legally excused) reflects a jurisprudential commitment to ease and compassion, reaffirming the Qur’anic assurance that hardship is not divinely intended (Qur’an 2:286).

The spiritual dimension of illness is further supported by the work of Lala et al,^7^ who described hardship as a catalyst for spiritual growth. Similarly, patients experiencing the physical and emotional challenges of catheterization face trials that test both medical resilience and spiritual outlook. By applying jurisprudential rulings that preserve dignity and facilitate worship, such challenges may be transformed into opportunities for strengthening faith and reliance upon Allah.

Consistent with Malkan et al,^8^ who demonstrated the Qur’an’s role in alleviating psychological distress, our study shows that clarity regarding purification rulings significantly reduces anxiety and spiritual uncertainty.

The Qur’anic injunction in Surah al-Mā’idah (5:6)^9^ establishes purification as a prerequisite for prayer while explicitly granting concessions through *tayammum* in cases of illness or hardship. This dual emphasis on obligation and facilitation directly informs the management of catheterized patients. Clinical–fiqh rulings permitting time-bound wuḍūūʾ or *tayammum* align with this verse, ensuring continuity of worship without imposing harm.

Afifi et al.^10^ further highlighted that purification in Islam serves both spiritual and hygienic purposes, particularly during public health crises. Jurisprudential concessions, therefore, complement medical safety while preserving religious obligations.

Previous scholarship addressing ritual purity in contexts such as menstruation and postpartum states emphasizes the necessity of alternative rulings to maintain religious participation. Osim and Eteng et al.^11^ highlighted these challenges among women, and similar considerations apply to catheterized patients, who likewise require adapted rulings to ensure inclusion and dignity.

The Prophetic statement, *“Purity is half of faith”* (Sahih Muslim)^12^ underscores the centrality of cleanliness in Islamic worship. In medical contexts where standard purification is difficult, jurisprudential flexibility ensures that patients are not spiritually marginalized due to illness or medical intervention.

Recent studies have also explored the intersection between ritual purification and hygiene. Akshija et al.^13^ suggested that ablution may contribute to microbial regulation, reinforcing the harmony between religious practice and health. Its adapted application in catheterized patients aligns with infection-prevention principles.

The Prophetic warning regarding negligence in protection from urine^14^,^15^ highlights the seriousness of purification in normal circumstances. However, Islamic jurisprudence clearly distinguishes between avoidable negligence and unavoidable medical conditions. Patients with continuous urinary drainage are therefore recognized as *ma‘dhūr*, allowing modified rulings that preserve worship without undue hardship.

Kitota et al.^16^ demonstrated that Prophetic teachings on hygiene closely align with modern public health principles. Jurisprudential accommodations thus support both spiritual obligations and clinical safety.

Finally, Kudhori et al.^17^ emphasized inclusivity and facilitation within Islamic law for individuals with disabilities. This principle directly applies to patients with indwelling catheters, affirming that Islamic jurisprudence adapts to medical realities while preserving religious practice.

## CONCLUSION

Muslim patients with indwelling urinary catheters should not discontinue prayer due to concerns regarding ritual purity. Islamic jurisprudence provides well-established concessions (rukhsah) for individuals classified as ma‘dhūr, allowing continuity of worship through time-bound ablution (wuḍūūʾ) or tayammum when medically indicated. The findings of this study demonstrate that counseling which integrates clinical realities with fiqh-based guidance significantly improves patient awareness, confidence, and spiritual reassurance.

Although issues of ritual purity extend to various medical interventions, Foley catheterization presents a unique challenge because of its direct association with continuous urinary drainage. Addressing this concern is therefore particularly important for preserving both patient dignity and religious well-being. Structured education based on Islamic legal principles can prevent unnecessary anxiety and ensure that patients do not omit obligatory worship due to misunderstanding.

The study supports the incorporation of faith-sensitive counseling into routine hospital care. Collaboration between healthcare institutions, Islamic educational bodies, and community organizations may facilitate the dissemination of clear, practical guidance through visual aids, educational materials, and public awareness initiatives. Such efforts would empower patients and caregivers with reliable information, enabling the maintenance of religious practice within the limits of medical feasibility.

### Take-Home Message:

Patients with indwelling urinary catheters remain obliged to perform ṣalāh according to their physical capacity—standing, sitting, or lying down. Continuous urinary drainage does not invalidate prayer once wuḍūūʾ or tayammum has been performed for the relevant prayer time. If the use of water poses medical risk or undue hardship, tayammum is permissible. The presence of a catheter does not exempt a patient from worship, and Islamic law provides facilitative rulings to ensure that medical treatment does not become a barrier to spiritual practice.

### Authors Contribution:

**WGB:** Conceived and designed the study, supervised the clinical component, performed data analysis, drafted and edited the manuscript, and is responsible for the integrity of the research.

**MG:** Contributed to the Islamic jurisprudential (fiqh) review, development of the clinical guidance framework, and critical revision of the manuscript for important intellectual content.

**UUS:** Assisted in participant recruitment, implementation of the structured guidance intervention, and manuscript drafting.

**ARK:** Contributed to literature review, data validation, and final critical review of the manuscript.
